# Identification and Purification of Human Induced Pluripotent Stem Cell-Derived Atrial-Like Cardiomyocytes Based on Sarcolipin Expression

**DOI:** 10.1371/journal.pone.0101316

**Published:** 2014-07-10

**Authors:** Rebecca Josowitz, Jia Lu, Christine Falce, Sunita L. D’Souza, Meng Wu, Ninette Cohen, Nicole C. Dubois, Yong Zhao, Eric A. Sobie, Glenn I. Fishman, Bruce D. Gelb

**Affiliations:** 1 The Mindich Child Health and Development Institute, Icahn School of Medicine at Mount Sinai, New York, New York, United States of America; 2 The Leon H. Charney Division of Cardiology, New York University School of Medicine, New York, New York, United States of America; 3 Department of Pharmacology and Systems Therapeutics, Icahn School of Medicine at Mount Sinai, New York, New York, United States of America; 4 Department of Developmental and Regenerative Biology, Icahn School of Medicine at Mount Sinai, New York, New York, United States of America; 5 Department of Genetics and Genomic Sciences, Icahn School of Medicine at Mount Sinai, New York, New York, United States of America; 6 Department of Medicine, New York University School of Medicine, New York, New York, United States of America; 7 Pediatrics, Icahn School of Medicine at Mount Sinai, New York, New York, United States of America; Merck & Co., United States of America

## Abstract

The use of human stem cell-derived cardiomyocytes to study atrial biology and disease has been restricted by the lack of a reliable method for stem cell-derived atrial cell labeling and purification. The goal of this study was to generate an atrial-specific reporter construct to identify and purify human stem cell-derived atrial-like cardiomyocytes. We have created a bacterial artificial chromosome (BAC) reporter construct in which fluorescence is driven by expression of the atrial-specific gene sarcolipin (*SLN*). When purified using flow cytometry, cells with high fluorescence specifically express atrial genes and display functional calcium handling and electrophysiological properties consistent with atrial cardiomyocytes. Our data indicate that *SLN* can be used as a marker to successfully monitor and isolate hiPSC-derived atrial-like cardiomyocytes. These purified cells may find many applications, including in the study of atrial-specific pathologies and chamber-specific lineage development.

## Introduction

The ability to differentiate human pluripotent stem cells into cardiomyocytes is a promising strategy for understanding human cardiac biology and disease [1]. Most stem cell-based studies modeling cardiac disease [2], [3] or drug responses [4], [5] have used mixed populations of cardiomyocytes. To date, it has been impossible to model atrial-specific disorders, study atrial-specific drug responses, or monitor *in* *vitro* atrial lineage specification, as there has been no way to reliably label and purify stem cell-derived atrial-like cardiomyocytes. Most atrial-associated genes, such as *MYL7* and *ANP*, are expressed in regions outside the atria during early development or even at maturity [6]. Accordingly, *MYL7* expression has been detected in ventricular-like populations of immature stem cell-derived cardiomyocytes [7]. The expression of one gene, sarcolipin (*SLN),* an inhibitor of the sarcoplasmic reticulum Ca^2+^-ATPase (SERCA), is restricted to the atrial lineage in the developing mouse heart from the onset of its expression, and this pattern is conserved in other mammals including humans [8]–[10]. However, it is unknown if *SLN* expression can be used to discriminate human atrial cells in differentiating pluripotent stem cell cultures, and if derived *SLN-*expressing cells would resemble functional atrial cardiomyocytes. To address these questions, we generated a bacterial artificial chromosome (BAC) reporter construct in which tdTomato fluorescence is driven by the expression of *SLN,* and evaluated its utility in differentiating hiPSC-derived cardiomyocytes.

## Materials and Methods

### Reporter line availability

Researchers interested in obtaining the reporter line should forward requests to the corresponding author.

### BAC recombineering and electroporation

The tdTomato reporter construct, encoding 1.4 kb tdTomato cDNA, 632 bp *Rex1* promoter, and 801 bp Neo^R^ gene was generated using standard cloning techniques. The reporter construct was recombineered into human BAC CTD-2651C21 (Invitrogen) as previously described [11]. Briefly, recombineering was performed in two steps. In the first step, 250 ng galK PCR product flanked by 50 bp homology arms located directly upstream and downstream of the *SLN* ATG start site was electroporated into electrocompetent SW102 cells harboring the BAC. Positive clones were obtained by selection on galactose-containing agar and verified by PCR. In the second step, the galK gene was replaced with the tdTomato reporter construct by electroporating 215 ng of the reporter construct (PCR product) flanked by 500 bp homology arms located directly upstream and downstream of the *SLN* ATG start site. Positive clones were obtained by selection on M63 minimal media plates with DOG and verified by PCR.

For electroporation of hiPSCs, recombineered BAC DNA was purified from DH10B cells using the Nucleobond BAC 100 kit (Macherey-Nagel) according to manufacturer’s instructions. Electroporation was performed as previously described [12] with the following modifications. hiPSCs were grown on matrigel-coated tissue culture dishes to 80% confluence. Cells were trypsinized and resuspended as single cells in hESC media. 50 µg purified BAC DNA was added to 10 million hiPSCs in a chilled 4 mm cuvette and incubated on ice for 5 min. Cells were electroporated using 320 V and 200 µF (BioRad), washed 1x with warmed hESC media, and plated on Neomycin-resistant MEFs (GlobalStem) in hESC media with 10 µM Y-27632 (Stemgent). After 2 days, clones were exposed to G418 25 µg/ml (Invitrogen). After 14 days, selection was increased to G418 50 µg/ml. Surviving clones were picked and verified by PCR (**Figure S1b**). Primers for verification are listed in **Table S2**.

### hiPSC generation and maintenance

Wild-type human dermal fibroblasts (Invitrogen) were reprogrammed using the mRNA Reprogramming Kit and Stemfect RNA Transfection Kit along with the microRNA Booster Kit (Stemgent) according to manufacturer’s instructions, with the following modifications. Fibroblasts (5×10^4^) were plated on matrigel-coated wells in DMEM/10% FCS media containing B18R supplement (Day -1). After 24 h, media was aspirated and fibroblasts were pre-incubated for 2–4 h with 2 mL fresh NuFF conditioned media containing 4 ng/ml pluriton supplement and 300 ng/ml B18R supplement. Fibroblasts were transfected with 3.5 µl/well of miRNA in Stemfect reagent and transfection buffer (Day 0). After 24 h media was changed to fresh NuFF conditioned media supplemented with 4 ng/ml pluriton supplement and 300 ng/ml B18R. Cells were transfected with 1 µg/well of mRNA cocktail in Stemfect transfection buffer (Day 1). This procedure was repeated for the following three days. On Day 5, the procedure from Day 0 was repeated. From Day 6–11, the procedure from Day 1 was repeated. On Day 12, media was changed to fresh NuFF conditioned media supplemented with 4 ng/ml pluriton supplement and 300 ng/ml B18R. After Day 14 single colonies were picked and expanded for pluripotency verification.

hiPSCs were maintained on mitotically inactivated MEFs in human ESC medium composed of DMEM/F12 (Cellgro, Mediatech) containing 20% (vol/vol) KSR (Invitrogen), 5% (vol/vol) MEF-conditioned medium, penicillin/streptomycin, L-glutamine (L-Gln), non-essential amino acids (Invitrogen), β-mercaptoethanol (β-ME, Sigma) and bFGF (R&D Systems). Pluripotency of transgenic hiPSCs was verified by immunofluorescence for OCT4, TRA1-81, and SSEA-4, and gene expression of *REX1* and *NANOG*. Alkaline phosphatase expression was verified using the Alkaline Phosphatase Staining Kit (Stemgent).

### Karyotyping

Karyotype analysis of G-banded metaphase chromosomes was performed at the Cytogenetics and Cytogenomics Laboratory at the Icahn School of Medicine at Mount Sinai. Transgenic hiPSCs were plated on matrigel-coated glass cover-slip dishes (MatTek), and karyotyping was performed as previously described [3].

### *In vitro* three germ layer differentiation

Transgenic hiPSCs were differentiated into endoderm, mesoderm, and ectoderm lineages *in* *vitro* using the d-Stem Tri-lineage Differentiation Kit (MicroStem) according to manufacturer’s instructions. In brief, 5×10^4^ hiPSCs were plated per chamber in 200 µl volume. After 24 h, Day 1 differentiation media for the three lineages was added to the respective chambers. Chambers were maintained for three days (mesoderm) or five days (endoderm, ectoderm) at 37°C in 5% CO_2_, 5% O_2_, and 90% N_2_ before fixation in 4% PFA for 15 min at room temperature. Chambers were washed with PBS and blocked for 1 h at room temperature in 3% milk, 1% BSA, and 0.1% TritonX100 in PBS. hiPSCs were stained with provided primary antibodies Brachyury/T (mesoderm), SOX17 (endoderm), or SOX1 (ectoderm) at 1∶200 dilution for 2 h at room temperature, followed by corresponding secondary antibody - AlexaFluor 488 goat-anti-rabbit IgG (mesoderm), goat-anti-mouse IgG (endoderm), donkey-anti-goat IgG (ectoderm) (Invitrogen) at 1∶400 dilution for 1 h at room temperature.

### hiPSC differentiation

hiPSCs were differentiated along a cardiac lineage as previously described [13] with the following modifications. Briefly, hiPSCs were passaged onto matrigel-coated plates and cultured for 2–3 days for feeder depletion. To generate EBs, hiPSCs were treated with 1 mg/ml collagenase B (Roche) for 15 min, and collected by gentle scraping. Cell clumps were centrifuged at 200 g for 2 min, and resuspended to small clusters of 50–100 cells by gentle pipetting in differentiation media containing StemPro 34 (Invitrogen), 2 mmol/L L-glutamine (Invitrogen), 4×10^4^ monothioglycerol (MTG, Sigma), 50 µg/ml ascorbic acid (Sigma), and 150 µg/ml transferrin (Roche). Differentiation media was supplemented with 10 ng/ml BMP4 (R&D Systems) (Day 0). EBs were maintained in 6-well ultra-low attachment plates (Corning) at 37°C in 5% CO_2_, 5% O_2_, and 90% N_2_. On Day 1, media was changed to differentiation media supplemented with 10 ng/ml BMP4 (R&D Systems) and 15 ng/ml Activin A (Peprotech). On Day 4, media was changed to differentiation media supplemented with 1.5 µmol/L IWR-1 [14] (Sigma) (**Figure S2**). After Day 8, media was changed every 5 days to differentiation media without supplements.

### Flow cytometry

For flow cytometric analyses, EBs were dissociated overnight in 1 mg/ml collagenase B (Roche) at 37°C, followed by incubation in TrypLE (Invitrogen) the next morning for 10–15 min to break up remaining EBs. To stain total cardiomyocytes, cells were stained with 1∶500 anti-human SIRPα-PE/Cy7 (BioLegend) and 1∶250 anti-human CD90-FITC (BD Pharmingen) for 1 h at 4°C in PBS/10% FBS staining buffer. Cells were filtered through a 40-µm cell strainer (Fisher) and resuspended at 10^6^ cells/mL in staining buffer for cell sorting. Sorting was performed on an AriaII cell sorter (BD Biosciences). Flow cytometric gates were set using control cells stained with the appropriate isotype control antibody. To determine cardiomyocyte purity, dissociated single cells were fixed with 4% PFA for 15 min at room temperature. Cells were then blocked in 2% BSA, 2% FBS, and 0.01% Triton for 1 h at room temperature. The primary antibody mouse-anti-human cTNT (ThermoScientific, clone 13–11) was conjugated to AlexaFluor 488 *in* *vitro* using the Zenon Mouse IgG Labeling Kit (Invitrogen), according to manufacturer’s instructions. Conjugated primary antibody was added to blocking solution at 1∶100 final dilution of cTNT antibody for 2 h at room temperature. Cells were analyzed on an LSR-II (BD Biosciences). Data were analyzed using FlowJo software, Version 9.3.2.

### Immunocytochemistry

Single cardiomyocytes or hiPSCs were cultured on matrigel-coated tissue culture plates (Falcon), and subsequently fixed in 4% PFA for 15 minutes at room temperature. Cells were blocked and permeabilized in 2% BSA, 2% FBS, and 0.01% Triton for 1 h at room temperature. Primary antibodies anti-human cTNT (1∶100, ThermoScientific), MLC2v (1∶200, Synaptic Systems), OCT4 (1∶100, BioVision), and SSEA4 (1∶25, Developmental Studies Hybridoma Bank) were added to blocking/permeabilization buffer overnight at 4°C. Secondary antibodies were either goat-anti-mouse or anti-rabbit AlexaFluor 488 or 594 (1∶400, Invitrogen). For TRA1-81 live cell immunostaining, hiPSCs were incubated with anti-human TRA1-81-Biotin (1∶100, eBioscience) for 2 h at 37°C in hESC media. Primary antibody was detected by incubation with PE-conjugated streptavadin secondary antibody (1∶100, eBioscience) for 2 h at 37°C in hESC media. Fluorescence was detected on the EVOS FL digital inverted fluorescent microscope (Life Technologies).

### Gene expression analysis

Total RNA was extracted using Trizol (Invitrogen) and RNeasy plus mini kit (Qiagen) from hiPSCs, EBs, and sorted red^high^ and red^low^ cardiomyocytes. Total RNA was reverse transcribed using oligo-dT primers with the Superscript II Synthesis Kit (Invitrogen). qPCR was performed using Fast SYBR Green Master Mix (Applied Biosystems) according to the manufacturer’s instructions. Expression levels were calculated using the ΔΔCT method and normalized to *GAPDH*. Real time qPCR was performed on a StepOne Plus Real-Time PCR System (Applied Biosystems) and analyzed with the StepOne Software v2.2.2. Primers and conditions used in qPCR assays are listed in **Table S2**. Normal human fetal heart RNA was purchased from Clontech (#636156), and normal human adult left atrium cDNA was purchased from Biochain (#A304014).

### Calcium transients and beat rate analysis

Sorted red^high^ and red^low^ cardiomyocytes were plated as single cells on matrigel-coated coverslips. After seven days, the cells were loaded with 10 µmol/L fluo-3 AM (Biotium) for 30 min at room temperature, then washed and superfused with Tyrode’s solution containing (mmol/L): NaCl 140, KCl 5.4, HEPES 10, NaH_2_PO_4_ 1, MgCl_2_ 1, CaCl_2_ 2, glucose 5 (pH 7.4). Fluo-3 AM was excited at 488 nm, and fluorescence above 505 nm was recorded by a confocal microscope (LSM 5 Exciter Carl Zeiss AG, Jena, Germany) at 40x magnification. Calcium transients were recorded from spontaneously beating myocytes using the line-scan mode of the microscope. Experiments were performed on a heated stage at 37°C. Analysis of data from the obtained line scan recordings consisted of: (1) averaging across the cell length, and (2) normalizing to fluorescence prior to stimulation (F/F0: relative fluorescence in arbitrary units). Calcium transient decay time constants were calculated by exponentially fitting functions to the declining phase of the transient. Recordings were processed and analyzed using custom MATLAB scripts.

The spontaneous beating rate of sorted red^high^ and red^low^ cardiomyocytes was determined optically by counting the number of beats per minute in bright-field mode of an inverted light microscope.

### Electrophysiology

Sorted red^high^ and red^low^ cardiomyocytes were re-cultured on matrigel-coated 35 mm tissue culture dishes. Dishes were transferred into a recording chamber within 48 to 72 h after plating for patch clamp studies. Cells were superfused with Tyrode’s solution containing (mmol/L) NaCl 137.7, KCl 5.4, NaOH 2.3, CaCl_2_1.8, MgCl_2_ 1, glucose 10, and HEPES 10 (pH adjusted to 7.4 with NaOH) at room temperature. Electrodes were filled with (mmol/L): KCl 50, K-aspartic acid 80, MgCl_2_ 1, EGTA 10, HEPES 10 and Na_2_-ATP 3 (pH adjusted to 7.2 with KOH). The liquid junction was ∼11 mV. The resistances of the electrodes were between 2 to 3 MΩ. Whole cell configuration was performed and only cells with gigaseal were used to collect data. Stimulated action potentials were triggered by minimum positive pulses with 1 Hz frequency with current clamp mode. For K^+^ current recording, 1 mmol/L BaCl_2_ and 0.2 mmol/L CdCl_2_ were used to block I_K1_ and I_Ca_ currents. Cells were held at −50 mV and prepulsed to +40 mV for 1 s to inactivate I_to_, followed by 160 ms test pulses between −40 to +50 mV. HCN4 currents were recorded by holding at −40 mV followed by a pulse to −140 mV. Signals were recorded by amplifiers (MultiClamp 700B, Axon Instruments Inc.) and digitized (Model DIGIDATA 1440A, Axon Instruments). Data acquisition and analysis were performed using CLAMPEX 10.2 and CLAMFIT 10.2 software (Axon instruments), respectively.

### Statistics

T-test was used for single comparisons. *P* values<0.05 were considered statistically significant. The number of stars indicates the significance level *<0.05, **<0.01, ***<0.001 and ****<0.0001. Data are presented as mean ± standard error of the mean.

## Results

### Generation and differentiation of transgenic hiPSCs

**Figure 1:**
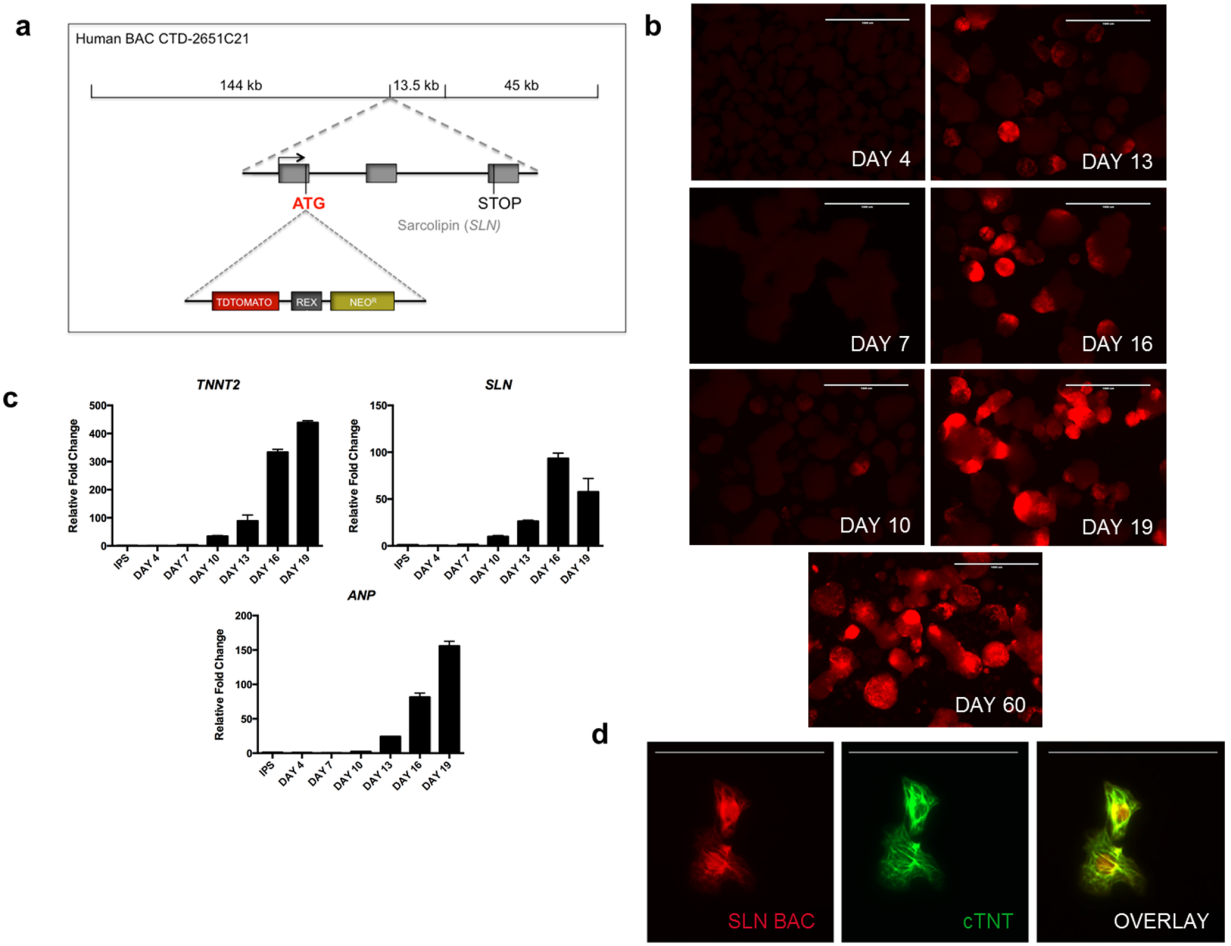
BAC transgene and expression in hiPSC-derived cardiomyocytes. (a) Schematic of recombineered BAC containing a tdTomato-*Rex1*-Neo^R^ cassette. (b) tdTomato fluorescence in differentiating EBs appeared at Day 10 of differentiation and increased over time, lasting up to 60 days. Scale bars, 1000 µm. (c) qPCR of differentiating EBs showing timeline of expression of *cTNT, SLN,* and *ANP* consistent with the appearance of red fluorescence. (d) Dissociated red^+^ cells were positive for cTNT by immunofluorescence. Scale bars, 100 µm.

To create the reporter construct, the human BAC (CTD-2651C21), encompassing the sequence 144 kb upstream of the human *SLN* start site, the *SLN* coding region, and 45 kb downstream, was used in order to maximize faithful *SLN* gene regulation. We utilized bacterial artificial chromosome recombineering techniques [11] to insert a tdTomato-*Rex1*-Neo^R^ cassette directly after the ATG start site (**Figure 1a**). Neomyocin resistance driven by the *Rex1* promoter enabled the selection of resistant transgenic hiPSC clones. The modified BAC was electroporated into wild-type hiPSCs and positive transgenic hiPSCs were selected by treatment with the gentamycin analog G418. Integration of the BAC was verified by PCR (**Figure S1a**), and pluripotency of the transgenic hiPSCs was verified *in* *vitro* (**Figure S1b–f**).

To assess whether the incorporated transgene could mark differentiating cardiac cells, hiPSCs were differentiated as embryoid bodies (EBs) along a cardiogenic lineage using a modified protocol of small molecule exposure over a series of days [13], [14] (**Figure S2**). Beating EBs appeared between Days 9–12 of differentiation. The onset of beating correlated with the appearance of areas of red fluorescence, which were first noticeable at Day 10 and increased over time, persisting up to 60 days (**Figure 1b**). Beating activity was always observed in red regions of EBs, but was also observed in non-red regions. Gene expression of cardiac troponin T type 2 (cTNT; *TNNT2)*, *SLN*, and *ANP* also appeared at Day 10 of differentiation and persisted over time, analogous to the timeline of appearance of red fluorescence (**Figure 1c**). We next dissociated beating EBs into single cells to analyze the phenotype of single tdTomato^+^ (red^+^) cells. Red^+^ cells displayed beating activity (**Video S1**), and stained positive for cTNT by both immunofluorescence and flow cytometry (**Figure 1d** **, Figure S3a**), validating their cardiac phenotype.

### Molecular characterization of purified red^high^ and red^low^ cardiomyocytes

**Figure 2:**
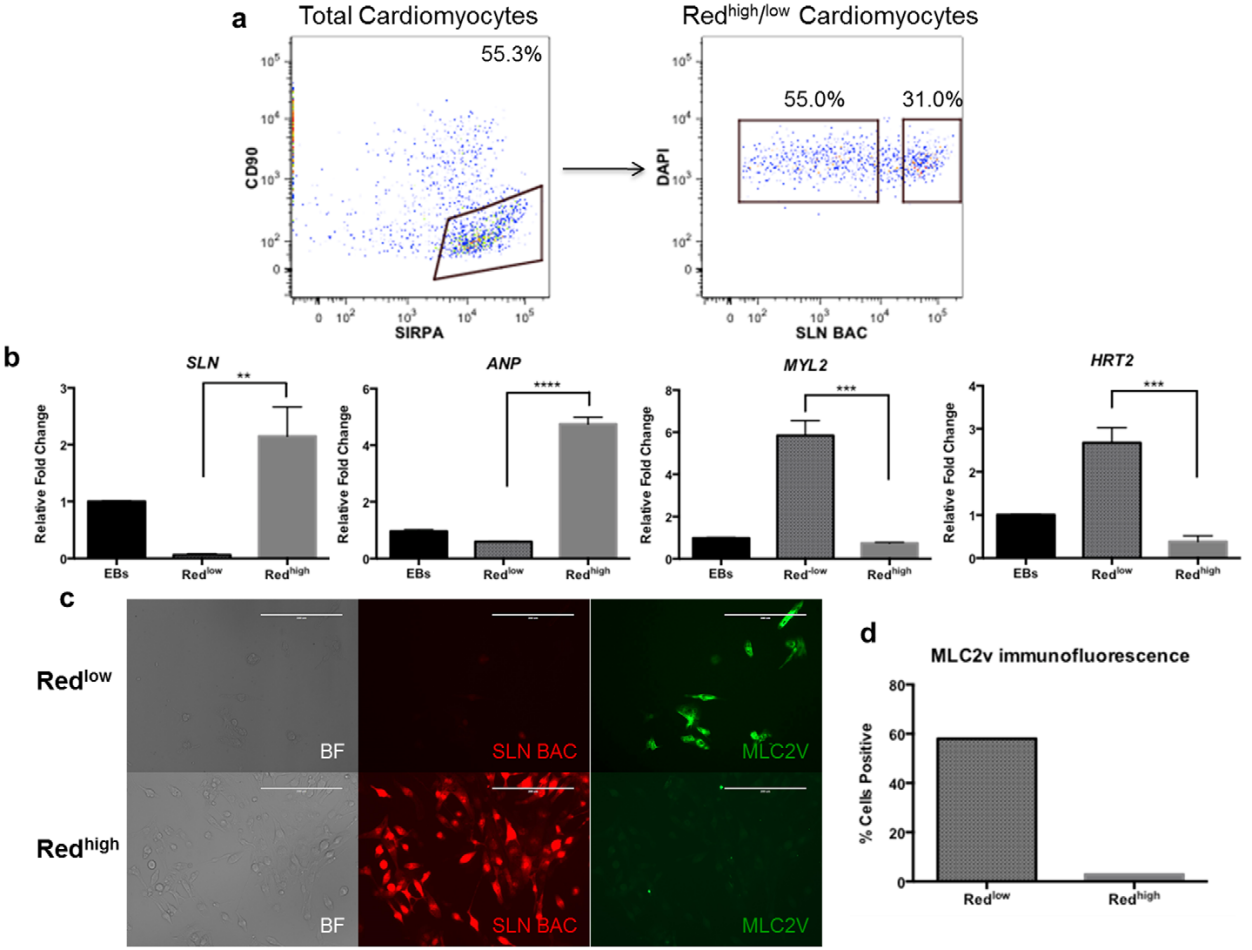
Purification and characterization of red^high^ and red^low^ cells. (a) Cell sorting strategy to purify red^high^ and red^low^ cardiomyocytes. Cells were first gated on the total cardiomyocyte population, marked as SIRPα^+^/CD90^−^, and then purified based on high or low levels of red fluorescence. (b) qPCR for relevant atrial (*SLN, ANP)* and ventricular (*MYL2, HRT2)* genes in purified red^high^ (n = 3) and red^low^ (n = 3) populations. (**p<0.01, ***p<0.001, ****p<0.0001). (c) Immunofluorescence for MLC2v showing exclusive expression in red^low^ population. Scale bars, 200 µm. (d) Quantification of fraction red^low^ (n = 143) and red^high^ (n = 514) cells expressing MLC2v protein from (c).

We next wanted to know whether red^+^ myocytes display an atrial-like phenotype in comparison to non-red cardiomyocytes. Importantly, co-staining dissociated EBs for cTNT demonstrated the existence of both cTNT^+^/red^+^ and cTNT^+^/red^−^ populations (**Figure S3a**). To maximize our ability to distinguish cardiomyocyte subtypes within the population of total cardiomyocytes using flow cytometry, we first stained dissociated EBs for SIRPα and CD90. SIRPα was recently identified as a surface marker for stem cell-derived cardiomyocytes [15], while CD90 is a surface marker specific for many non-cardiomyocyte cell types, including the majority of non-cardiomyocytes derived from pluripotent stem cells [15], [16] (**Figure S3c**). We gated on the total cardiomyocyte population (SIRPα^+^/CD90^−^), and then sorted the red high (red^high^) and red low (red^l^°^w^) populations within the total cardiomyocyte population **(Figure S3b)**. A wide range of red fluorescence intensity was observed in differentiated cardiomyocytes (**Figure 2a** **)**. This phenomenon is likely due to positional effects of random transgene integration, causing weak expression of the transgene in non-atrial cells, but could also be due to low levels of *SLN* expression in progenitor cells or populations of mixed maturity that do not yet display an atrial phenotype. Despite this, we consistently observed a very strongly red fluorescent sub-population. In order to simply establish whether strongly red^high^ cells are indeed atrial-like, we chose cell sorting gates conservatively, identifying red^high^ cells as those with very high red fluorescence, and red^low^ cells as those with low fluorescence. Based on these gates, red^high^ cells comprised ∼31% of the total cardiomyocyte population while red^low^ cells comprised ∼55%. Sorted red^high^ cells displayed significantly increased expression of both *ANP* and *SLN* compared to red^low^ cells (∼8- and 33-fold, respectively). In contrast, the red^high^ cells displayed significantly decreased expression of the ventricular-specific genes *MYL2* and *HRT2* compared to red^low^ cells (∼8- and 7-fold, respectively) (**Figure 2b**). Immunostaining for MLC2v confirmed expression restricted to the red^low^ population (**Figure 2c, d**).

### Purified red^high^ cells display calcium handling properties similar to atrial cardiomyocytes

**Figure 3:**
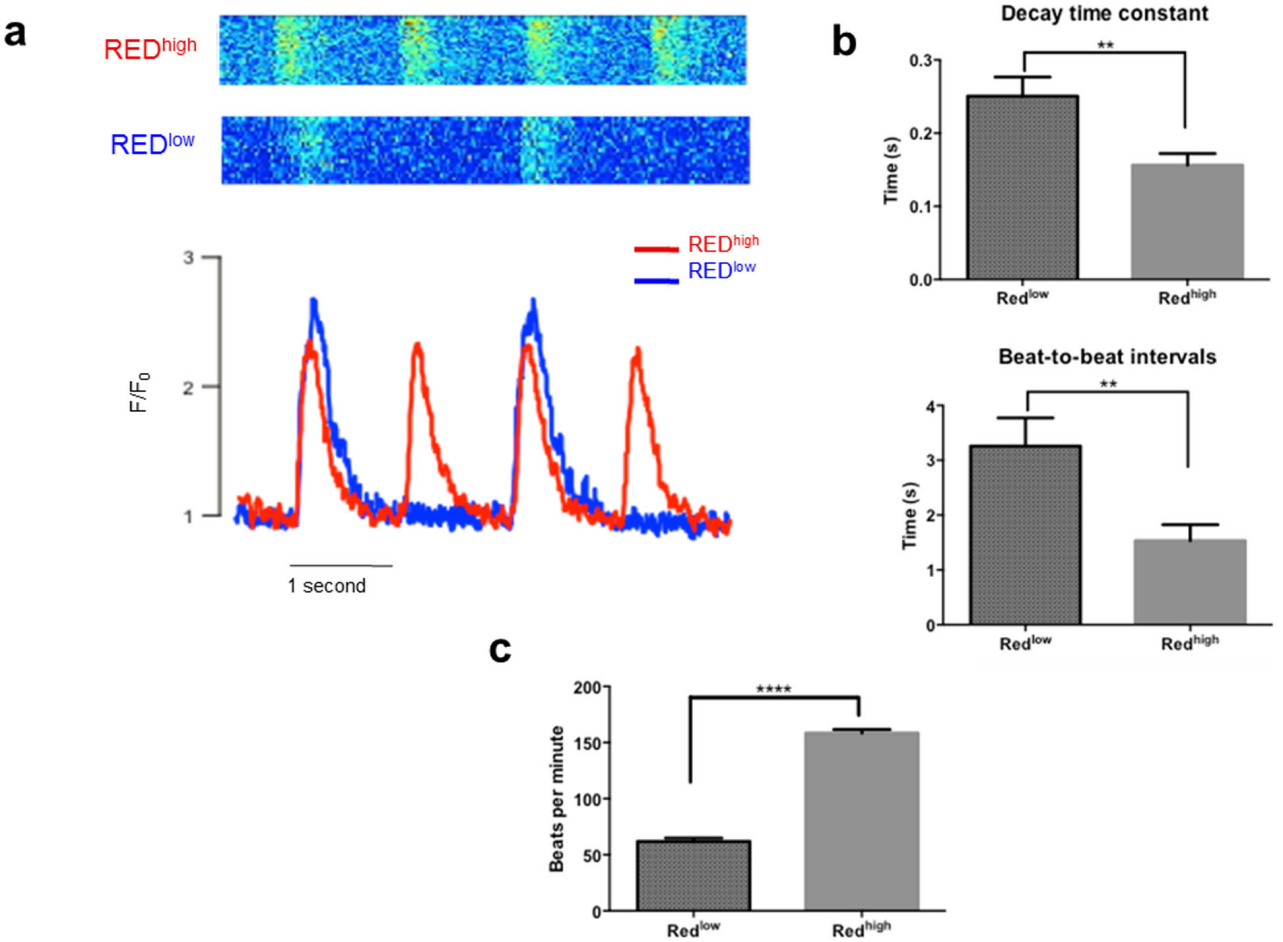
Red^high^ cells possess functional Ca^2+^ handling properties similar to atrial cardiomyocytes. (a) Representative spontaneous calcium transients recorded from red^high^ and red^low^ cells. (b) Quantification of calcium transient decay and interval properties in red^high^ (n = 13) and red^low^ (n = 13) cells (**p<0.01). (c) Quantification of beating rate in spontaneously beating red^high^ (n = 22) and red^low^ (n = 23) cells (****p<0.0001).

Next, we assessed the functional properties of purified red^high^ and red^low^ cardiomyocytes. Analysis of spontaneous calcium (Ca^2+^) transients revealed significantly shorter beat-to-beat intervals (1.5 s *vs* 3.2 s; p = 0.007) and time constants of Ca^2+^ transient decay (0.15 s *vs* 0.25 s; p = 0.004) in red^high^ cells compared to red^low^ cells (**Figure 3a, b**). These properties are consistent with faster spontaneous depolarization and the increased rate of sarcoplasmic reticulum Ca^2+^ re-uptake documented in rat atrial myocytes [17]. To confirm whether more frequent spontaneous Ca^2+^ transients in red^high^ cells correlated with more frequent contractions, sorted cells were re-cultured and the spontaneous beating rate was quantified. Indeed, red^high^ cells beat significantly faster than red^low^ cells (158 bpm *vs* 62 bpm; p<0.0001) (**Figure 3c**), consistent with the more rapid spontaneous depolarization of atrial myocytes compared to ventricular myocytes. Although there are well-appreciated differences in t-tubule organization between atrial and ventricular cardiomyocytes [18], staining with a lipophilic membrane dye did not reveal the presence of t-tubules in red^high^ or red^low^ cardiomyocytes, consistent with their immature state as previously documented [19] (data not shown).

### Purified red^high^ cardiomyocytes display atrial-like electrophysiological properties

**Figure 4:**
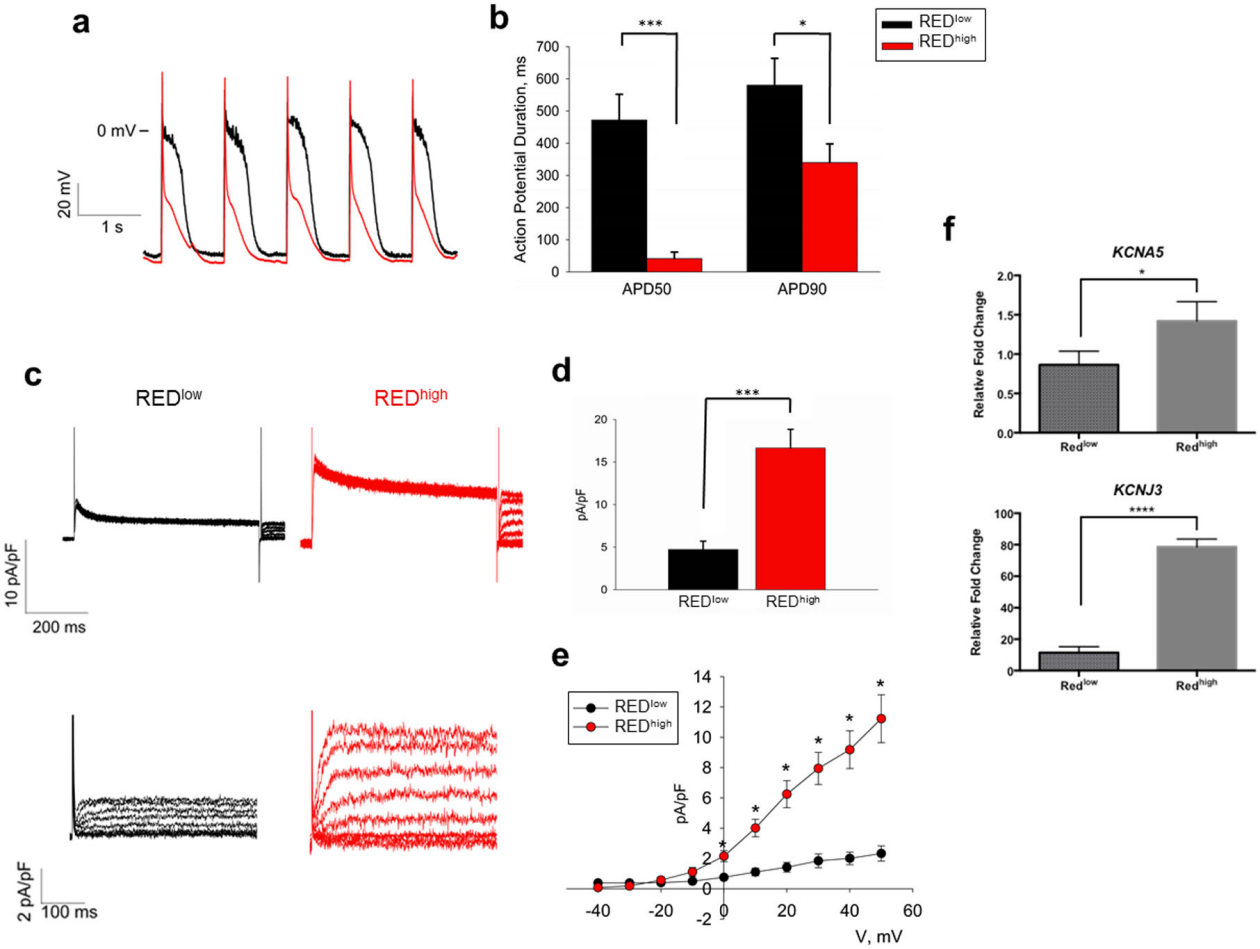
Red^high^ cells display electrophysiological properties similar to atrial cardiomyocytes. (a) Representative triggered action potential traces recorded from red^high^ and red^low^ cells. (b) Quantification of APD_50_ and APD_90_ for red^high^ (n = 15) and red^low^ (n = 10) cells (***p<0.001, *p<0.05). (c–e) Expression of depolarization-activated potassium currents. (c) Upper panel: representative instantaneous outward currents. Lower panel: representative sustained potassium currents. (d) Comparison of peak instantaneous outward currents between red^high^ (n = 7) and red^low^ (n = 8) cells. (e) Comparison of I–V curves of sustained potassium currents between red^high^ and red^low^ cells (***p<0.001). (f) qPCR for gene expression of the I_Kur_ subunit *KCNA5* and I_KAch_ subunit *KCNJ3*, revealing increased expression in red^high^ cells (*p<0.05, ****p<0.0001). All genes normalized to expression of *GAPDH* and relative to gene expression in EBs.

Electrophysiological assessment revealed action potentials (APs) from red^high^ and red^low^ cells to be characteristic of atrial and ventricular myocytes, respectively (**Figure 4a, b**). APs from red^high^ cells paced at 1 Hz at room temperature had a fast up-stroke and down-stroke lacking a plateau phase, and average APD_50_ and APD_90_ of 42 and 340 ms, respectively. In contrast, APs from red^low^ cells paced at 1 Hz displayed the distinct plateau phase of ventricular myocytes, and had a significantly more prolonged average APD_50_ and APD_90_ of 472 and 580 ms, respectively. Red^high^ and red^low^ cells displayed similar resting membrane potentials (red^high^ −70.4 mV, red^low^ −69.0 mV) and peak AP amplitudes (red^high^ 106 mV, red^low^ 102 mV) (**Table S1**). These properties are consistent with previous studies of atrial-like and ventricular-like hiPSC-derived cardiomyocytes which have been reported to possess resting membrane potentials around −70 mV and peak AP amplitudes of ∼100 mV [20]. We also assessed differences in outward potassium (K^+^) current in red^high^ cells compared to red^low^ cells (**Figure 4c–e**). The instantaneous outward K^+^ current is due to I_to_, which is activated within 10 ms. Voltage-clamp measurements revealed red^high^ cells displayed increased peak instantaneous outward K^+^ current compared with red^low^ cells, consistent with previous observations in isolated primary human atrial and ventricular cardiomyocytes [21]. Red^high^ cells also displayed increased sustained outward K^+^ current compared to red^low^ cells. The sustained outward K^+^ current comprises various K^+^ channels, including I_Kur_, I_Kr_, I_Ks_, and I_KAch_. While differences in I_Kr_ and I_Ks_ currents in atrial and ventricular cardiomyocytes are not well documented [22], I_Kur_ and I_KAch_ are highly enriched in atrial cardiomyocytes [23]. Accordingly, increased expression of *KCNA5*, a subunit of the I_Kur_ complex, and *KCNJ3*, a subunit of the I_KAch_ complex, was detected in red^high^ cells compared to red^low^ cells (**Figure 4f**), suggesting that the differential expression of atrial-specific potassium channels is conserved in differentiating stem cell-derived cardiomyocytes.

## Discussion

We have provided a proof-of-concept study to show that *SLN* expression can be used as a marker to successfully monitor and isolate hiPSC-derived atrial-like myocytes. *SLN* expression appears concurrent with the onset of beating, and continues for extended periods in culture, allowing for isolation of highly red fluorescent atrial-like cells at early or later time-points during differentiation. The purified atrial-like cardiomyocytes are functional and express known atrial-associated genes, including those encoding components of the I_Kur_ and I_KAch_ complexes, which contribute to their distinct AP properties.

Interestingly, we did not observe AP morphologies consistent with nodal-like cardiomyocytes in either the red^high^ or red^low^ population. Nodal-like myocytes have been reported to possess less hyperpolarized resting membrane potentials around −60 mV, smaller peak AP amplitudes of ∼80 mV, and slower upstroke velocities [20]. HCN4 current, responsible for the funny current normally restricted to mature pacemaker cells, is not an optimal marker for stem cell-derived nodal cells, as immature derived cardiomyocytes display persistent *HCN4* expression and spontaneous beating activity [24]. Accordingly, automaticity and HCN4 current was detected in both red^high^ and red^low^ cells (**Figure S4a**), and gene expression of *HCN4, ANP*, and *SLN* in red^high^ cells revealed more similarity to fetal heart than adult atrial samples (**Figure S4b**), suggesting an immature phenotype. Indeed, the resting membrane potentials found in red^high^ and red^low^ cells are more consistent with immature cardiomyocytes rather than their adult counterparts [25]. However, a comparative gene expression array may provide better clarity about the maturation state of the red^high^ and red^low^ cells. Our inability to detect nodal-like cells is likely due to their very low prevalence in our culture or the requirement for more time to develop a mature nodal phenotype *in* *vitro*.

Studies performed with mixed cardiomyocyte populations are not optimal, as there are well-documented differences in ion channel expression and function between human atrial and ventricular cardiomyocytes [26]. Our ability to purify stem cell-derived atrial-like cardiomyocytes will facilitate the study of specific atrial pathologies such as atrial arrhythmias. These purified atrial-like cells can also enhance our understanding of atrial biology, and perhaps find utility as a tool to discover novel atrial-specific cell surface markers. The ability to fluorescently monitor the differentiation of atrial-like cells over time will also facilitate our understanding of cardiac lineage specification. In the mouse heart, *SLN* transcript becomes detectable at E12.5 [9], immediately following atrial and ventricular septation beginning at E11.5, and concurrent with initiation of atrioventricular canal septation. Our ability to detect *SLN* expression beginning at Day 10 of differentiation may provide a relevant timeframe for the onset of lineage specification in hiPSC-derived cardiomyocytes. Interestingly, *SLN* transcript levels decrease from Day 16 to Day 19 of differentiation, indicating *SLN* expression may peak during earlier time points critical for atrial specification and decrease at later time points. However, we were still able to detect transgene expression and FACS sort a clear red^high^ population at Day 60 of differentiation.

By combining *SLN* transgenic markers with markers of other lineages or precursor populations such as *MYL2*, *ISL1*, or *TBX3* [27]–[29], we can better understand the genetic and cellular interactions underpinning cardiac development. Lastly, as our *in* *vitro* techniques and understanding of the potential for cardiac regeneration improve, hiPSC-derived cardiomyocytes will likely find enhanced clinical application in cell therapy. It will be imperative to utilize a defined population of cells to avoid unintended pathologies. Injection of heterogeneous cardiomyocytes with varying electrophysiological properties may have potential arrhythmic consequences [30], [31], while the use of pure atrial cells should be optimal for delivery to atrial tissue.

In summary, hiPSC-derived atrial-like cardiomyocytes identified by *SLN* expression may be a valuable tool to enhance our understanding of atrial biology and disease.

## Supporting Information

Figure S1 **Verification of BAC and hiPSC pluripotency.** (a) PCR amplification of two BAC regions from genomic DNA from G418 resistant hiPSCs, verifying integration. Gene product A obtained using tdTomato-F and Rex1-R primers. Gene product B obtained using Rex1-F and Neo^R^-R primers. Bands are the same as those amplified from the BAC itself. (b) Alkaline phosphatase staining of transgenic hiPSCs. (c) Immunofluorescence for pluripotency markers in transgenic hiPSCs. Cells were fixed and stained with anti-OCT4 and anti-SSEA4, or stained live with anti-Tra1-81. Scale bars, 400 µm. (d) Gene expression for pluripotency markers *NANOG* and *REX1*, indicating transgenic hiPSCs display similar gene expression levels to hES cells. All genes normalized to expression of *GAPDH* and relative to gene expression in hESC. (e) G-banding of transgenic hiPSC line demonstrates normal diploid chromosomes. (f) *In vitro* differentiation of transgenic hiPSCs into mesoderm, endoderm, and ectoderm lineages. Scale bars, 200 µm.
(PDF)

Figure S2 **Schematic of cardiac differentiation protocol.** hiPSCs were differentiated into cardiomyocytes by exposure of embryoid bodies to BMP4, Activin A, and IWR-1 over a series of days. Beating EBs appeared between Day 9–12 and were dissociated for analysis after Day 25.
(PDF)

Figure S3 **Flow cytometry sorting strategies.** (a) Identification of a cTNT^+^/red^+^ and cTNT^+^/red^−^ population. (b) Overlay of red^high^ population on population of total live cells, showing red^high^ cells comprise a portion of the total cardiomyocyte population. (c) Sorting for SIRPα^+^/CD90^−^, population enriches cardiomyocyte fraction >96%.
(PDF)

Figure S4 **Isolated red^high^ cardiomyocytes are fetal-like.** (a) Representative voltage-clamp recording of HCN4 current in both red^low^ and red^high^ cells. (b) Gene expression of *HCN4, SLN* and *ANP* suggests isolated red^high^ cardiomyocytes are more similar to fetal heart than adult left atrial tissue. All genes normalized to expression of *GAPDH* and relative to gene expression in EBs.
(PDF)

Table S1 **Action potential characteristics of red^high^ and red^low^ cardiomyocytes.**
(PDF)

Table S2 **Primers used in real-time qPCR and regular PCR experiments.**
(PDF)

Video S1 **Spontaneous beating activity in single red^+^ cells.**
(MOV)
