# Supplementary figures and images for: Identification and Purification of Human Induced Pluripotent Stem Cell-Derived Atrial-Like Cardiomyocytes Based on Sarcolipin Expression
Source: PLoS One. 2014 Jul 10;9(7):e101316. doi: 10.1371/journal.pone.0101316 (PMC4092021; doi:10.1371/journal.pone.0101316)

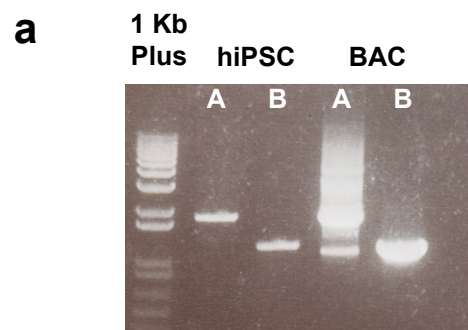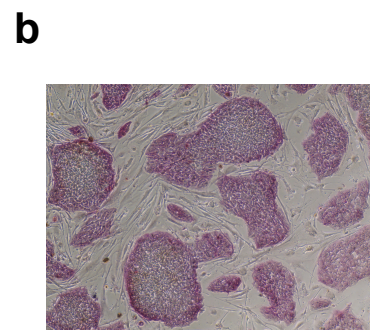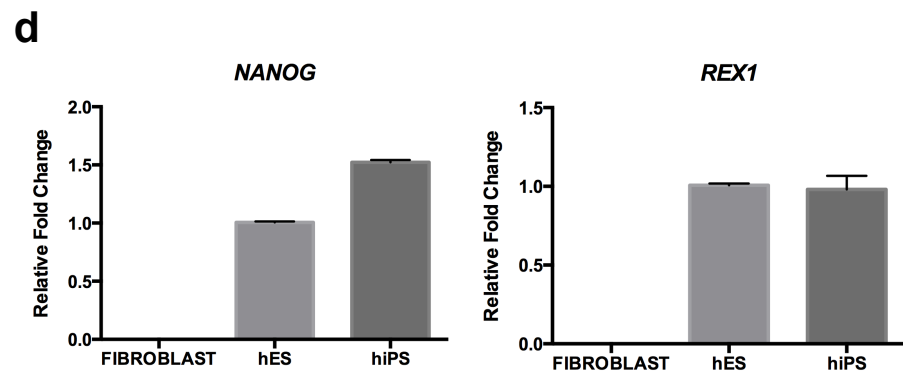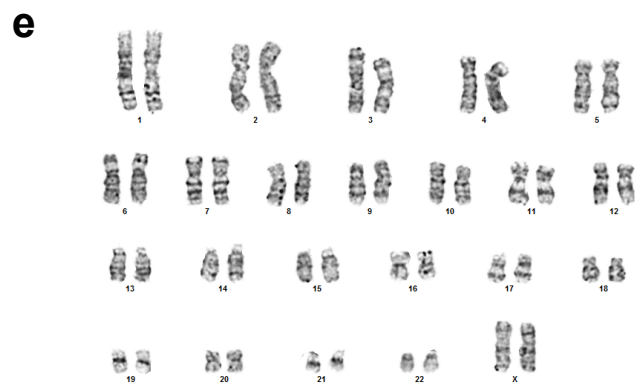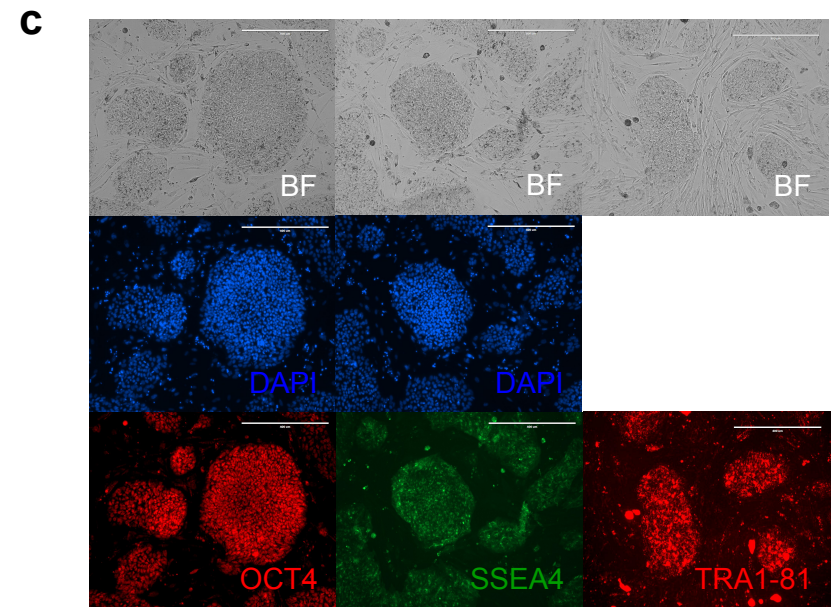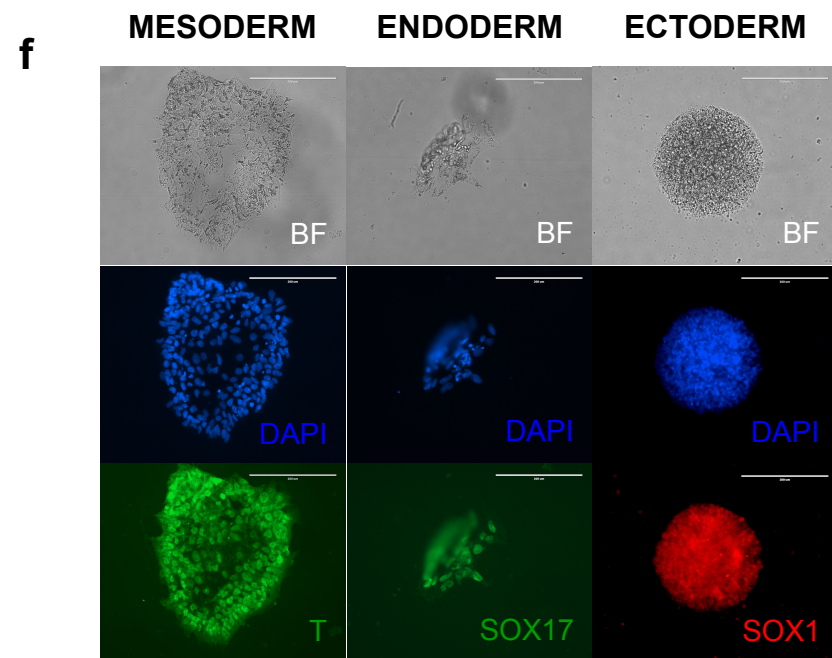

Supplement: Figure S1 — Verification of BAC and hiPSC pluripotency. (a) PCR amplification of two BAC regions from genomic DNA from G418 resistant hiPSCs, verifying integration. Gene product A obtained using tdTomato-F and Rex1-R primers. Gene product B obtained using Rex1-F and NeoR-R primers. Bands are the same as those amplified from the BAC itself. (b) Alkaline phosphatase staining of transgenic hiPSCs. (c) Immunofluorescence for pluripotency markers in transgenic hiPSCs. Cells were fixed and stained with anti-OCT4 and anti-SSEA4, or stained live with anti-Tra1-81. Scale bars, 400 µm. (d) Gene expression for pluripotency markers NANOG and REX1, indicating transgenic hiPSCs display similar gene expression levels to hES cells. All genes normalized to expression of GAPDH and relative to gene expression in hESC. (e) G-banding of transgenic hiPSC line demonstrates normal diploid chromosomes. (f) In vitro differentiation of transgenic hiPSCs into mesoderm, endoderm, and ectoderm lineages. Scale bars, 200 µm. (PDF) [file pone.0101316.s001.pdf]

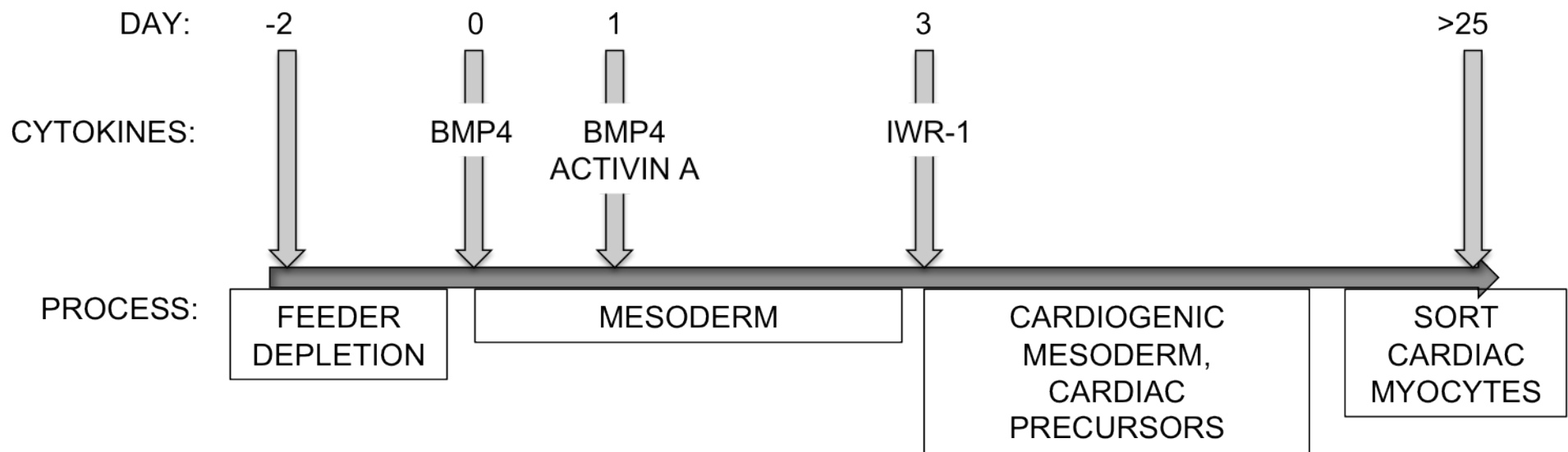

Supplement: Figure S2 — Schematic of cardiac differentiation protocol. hiPSCs were differentiated into cardiomyocytes by exposure of embryoid bodies to BMP4, Activin A, and IWR-1 over a series of days. Beating EBs appeared between Day 9–12 and were dissociated for analysis after Day 25. (PDF) [file pone.0101316.s002.pdf]

**a**

Unstained

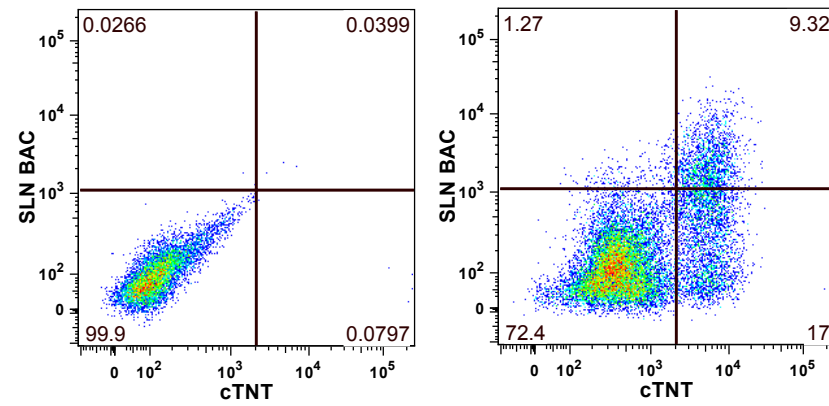**b**

Total Live Cells

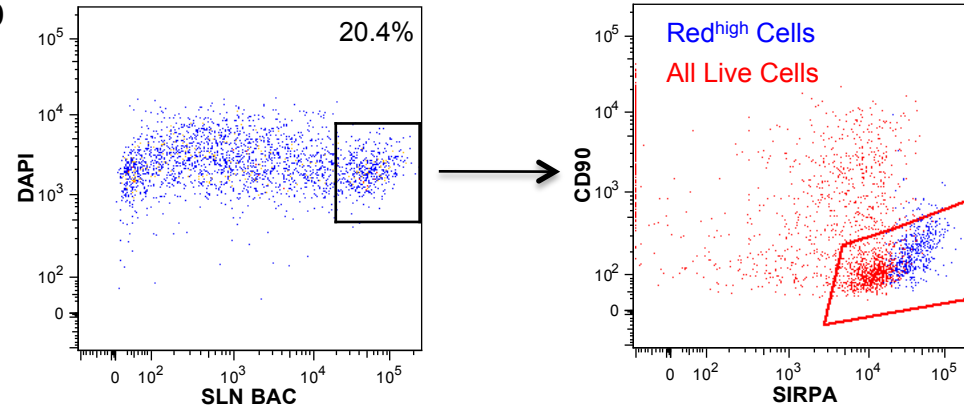**c**

Sorting

Pre-sort

Post-sort

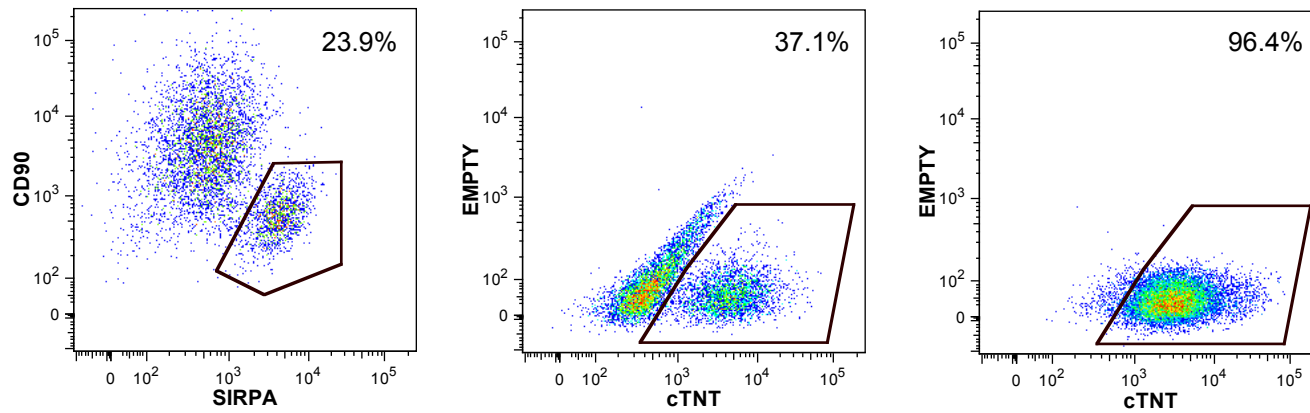

Supplement: Figure S3 — Flow cytometry sorting strategies. (a) Identification of a cTNT+/red+ and cTNT+/red− population. (b) Overlay of redhigh population on population of total live cells, showing redhigh cells comprise a portion of the total cardiomyocyte population. (c) Sorting for SIRPα+/CD90−, population enriches cardiomyocyte fraction >96%. (PDF) [file pone.0101316.s003.pdf]

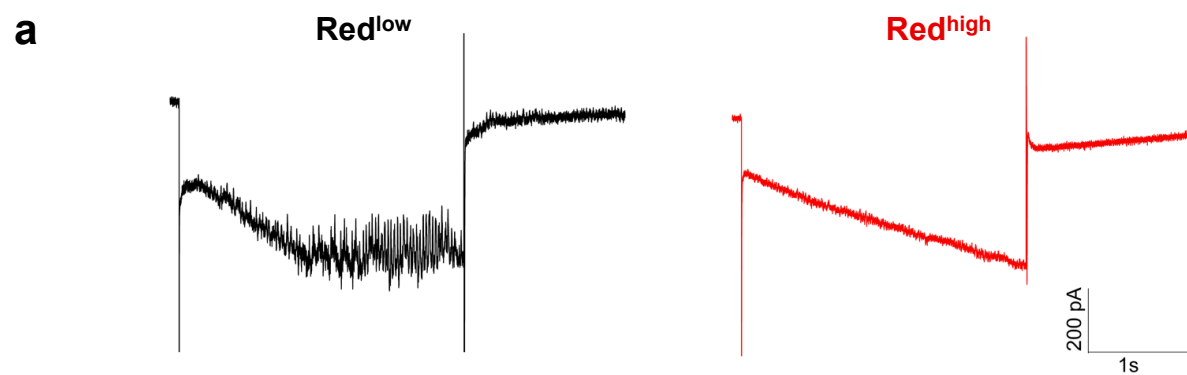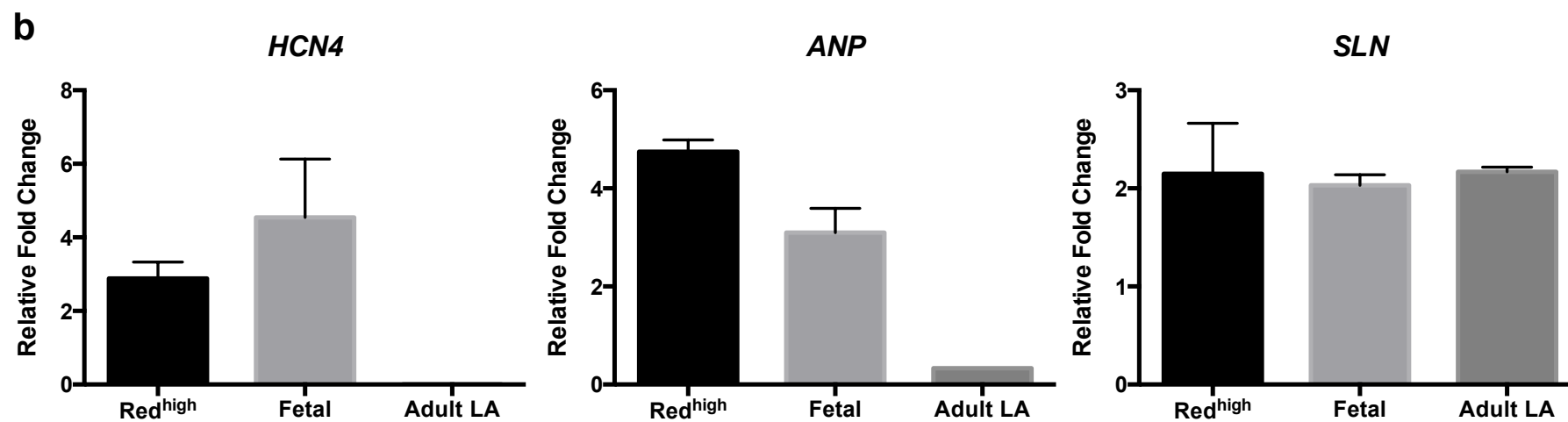

Supplement: Figure S4 — Isolated redhigh cardiomyocytes are fetal-like. (a) Representative voltage-clamp recording of HCN4 current in both redlow and redhigh cells. (b) Gene expression of HCN4, SLN and ANP suggests isolated redhigh cardiomyocytes are more similar to fetal heart than adult left atrial tissue. All genes normalized to expression of GAPDH and relative to gene expression in EBs. (PDF) [file pone.0101316.s004.pdf]
