# Supplementary material for: Identification and Purification of Human Induced Pluripotent Stem Cell-Derived Atrial-Like Cardiomyocytes Based on Sarcolipin Expression
Source: PLoS One. 2014 Jul 10;9(7):e101316. doi: 10.1371/journal.pone.0101316 (PMC4092021; doi:10.1371/journal.pone.0101316)
Supplement: Table S1 — Action potential characteristics of redhigh and redlow cardiomyocytes. (PDF) [file pone.0101316.s005.pdf]

	Resting Potential (mV)	AP Amplitude (mV)	APD50 (ms)	APD90 (ms)
Red^{high}	-70.4±1.3	106.3±2.9	41.5±20.2	339.9±57.8
Red^{low}	-69.0±1.5	101.9±2.2	472.0±79.9***	580.2±83.4*
