# Supplementary material for: Identification and Purification of Human Induced Pluripotent Stem Cell-Derived Atrial-Like Cardiomyocytes Based on Sarcolipin Expression
Source: PLoS One. 2014 Jul 10;9(7):e101316. doi: 10.1371/journal.pone.0101316 (PMC4092021; doi:10.1371/journal.pone.0101316)
Supplement: Table S2 — Primers used in real-time qPCR and regular PCR experiments. (PDF) [file pone.0101316.s006.pdf]

Gene	Forward Primer (5'-3')	Reverse Primer (5'-3')
QPCR		
NANOG	CCTGAAGACGTGTGAAGATGAG	GCTGATTAGGCTCCAACCATAC
REX1	AAAGCATCTCCTCATTCATGGT	AAAGCATCTCCTCATTCATGGT
cTNT	AGCATCTATAACTTGGAGGCAGAG	TGGAGACTTTCTGGTTATCGTTG
ANP	ACGCAGACCTGATGGATTTC	GCTTCTTCATTCGGCTCACT
SLN	CTTGGTGTGCCCTCAGAAAT	TCAGTCAATCCCAGGACCAT
MYL2	TACGTTCGGGAAATGCTGAC	TTCTCCGTGGGTGATGATG
HRT2	TTTGAAGATGCTTCAGGCAA	GGCACTCTCGGAATCCTATG
KCNA5	GAGGACGAGGAGGAAGAAGG	AGGAGTGTGTTGGGGAACTG
KCNJ3	GGACGGAAAACTCACGCTTA	TCAAGTTGGTCAAGGGGAAG
GAPDH	GAGTCAACGGATTTGGTCGT	TTGATTTTGGAGGGATCTCG
PCR		
TDTOMATO	GCGAGGAGGTCATCAAAGAG	
REX1	ACCGATTCCTCCCGATAAGT	CAGCGTTCACCACTGACTACA
NEO^{R}		GATGTTTCGCTTGGTGGTCG
